# Improving Micro-EDM Machining Efficiency for Titanium Alloy Fabrication with Advanced Coated Electrodes

**DOI:** 10.3390/mi15060692

**Published:** 2024-05-24

**Authors:** Hoang-Vuong Pham, Huu-Phan Nguyen, Shirguppikar Shailesh, Duc-Toan Nguyen, Ngoc-Tam Bui

**Affiliations:** 1 Faculty of Mechanical Engineering, University of Transport and Communications, No. 3, Cau Giay Street, Lang Thuong Ward, Dong Da District, Hanoi 100000, Vietnam; phvuong@utc.edu.vn; 2 Faculty of Mechanical Engineering, School of Mechanical and Automotive Engineering, Hanoi University of Industry, No. 298 Cau Dien Street, Bac Tu Liem District, Hanoi 100000, Vietnam; nguyenhuuphan@haui.edu.vn; 3 Department of Mechatronics Engineering, Rajarambapu Institute of Technology, Shivaji University, Kolhapur 414415, Sakharale, India; shailesh.shirguppikar@ritindia.edu; 4 School of Mechanical Engineering, Ha Noi University of Science and Technology, No. 1 Dai Co Viet Stress, Hai Ba Trung District, Hanoi 100000, Vietnam; 5 Innovative Global Program, Shibaura Institute of Technology, Tokyo 135-8548, Japan; tambn@shibaura-it.ac.jp

**Keywords:** Micro-EDM, coated electrode, surface quality, Ti-6Al-4V, efficacy

## Abstract

Enhancing the operational efficacy of electrical discharge machining (EDM) is crucial for achieving optimal results in various engineering materials. This study introduces an innovative solution—the use of coated electrodes—representing a significant advancement over current limitations. The choice of coating material is critical for micro-EDM performance, necessitating a thorough investigation of its impact. This research explores the application of different coating materials (AlCrN, TiN, and Carbon) on WC electrodes in micro-EDM processes specifically designed for Ti-6Al-4V. A comprehensive assessment was conducted, focusing on key quality indicators such as depth of cut (Z), tool wear rate (TWR), overcut (OVC), and post-machining surface quality. Through rigorous experimental methods, the study demonstrates substantial improvements in these quality parameters with coated electrodes. The results show significant enhancements, including increased Z, reduced TWR and OVC, and improved surface quality. This evidence underscores the effectiveness of coated electrodes in enhancing micro-EDM performance, marking a notable advancement in the precision and quality of Ti–6Al–4V machining processes. Among the evaluated coatings, AlCrN-coated electrodes exhibited the greatest increase in Z, the most significant reduction in TWR, and the best OVC performance compared to other coatings and the uncoated counterpart.

## 1. Introduction

The Ti-6Al-4V alloy is renowned for its robust mechanical properties, presenting formidable challenges to conventional machining methods. Electrical Discharge Machining (EDM) has emerged as a compelling alternative, particularly for fabricating micro-scale products. Within the micro-EDM domain, optimizing productivity and machining precision is crucial, driving ongoing research to enhance these quality metrics. Various technological innovations have been proposed to address the limitations of micro-EDM, including workpiece or electrode vibration modulation, integration of dielectric solution-borne powders, and the utilization of coated electrodes. Among these, the use of coated electrodes shows significant potential to improve quality parameters. The selection of suitable coating materials is critical for micro-EDM efficiency, relying on the electrical, thermal, and physico-chemical properties of the coatings. Additionally, the interaction between the workpiece material and micro-EDM process parameters is complex and must be carefully considered. Despite these challenges, there is a limited body of research on suitable coating materials for micro-EDM, warranting further investigation.

The surface composition of the electrode in micro-EDM impacts both the technical and economic aspects of the machining process. For example, research has shown that using copper (Cu)-coated aluminum (Al) electrodes can reduce machining costs by about one-third compared to Cu electrodes [1]. Despite the cost implications of coated electrodes, this factor has not been fully addressed in scholarly work. Examining quality parameters such as Material Removal Rate (MRR), TWR, and Surface Roughness (SR) in EDM with silver-coated Cu electrodes shows substantial improvements, making silver-based materials suitable for tool electrodes [2].

Furthermore, applying Al_2_O_3_–TiO_2_ coatings to tool electrodes significantly improves wear resistance and machining precision, achieving a 92% reduction in TWR and a 62.5% decrease in Overcut (OVC) [3]. Conversely, using Cu coating on WC electrodes in micro-EDM enhances MRR compared to Ag-coated materials [4], though it increases TWR. Analysis of machined surfaces using Cu-coated electrodes reveals the penetration of Cu and carbon into the layer, altering its physicochemical properties [5]. Quality parameters in EDM with MWCNT Cu-base coated electrodes show significant improvements, with the coating material present in the machined surface layer affecting mechanical, physical, and chemical properties.

Strategies involving Cu-coated Gr electrodes yield substantial improvements in SR [6], while Ni-coated electrodes in micro-EDM enhance MRR, TWR, and surface topography [7]. Comparative analyses of TiN, Ag, and ZrN-coated electrodes highlight the favorable attributes of TiN, which exhibits minimal Electrode Wear Rate (EWR) and OVC, in contrast to Ag [8]. TiN coating notably reduces TWR and OVC in micro-EDM with WC electrodes by approximately 16.32% and 26%, respectively, while increasing machining depth by about 18.9% [9]. These findings suggest that Cu-coated Al electrodes can reduce costs and weight compared to Cu electrodes [10].

The use of Cu–ZrB2 and ZnC coatings has been shown to significantly improve TWR [11]. The effectiveness of electrode coating materials in EDM depends on their intrinsic properties, such as melting point, electrical conductivity, and thermal conductivity [12]. Compared to uncoated electrodes, coated variants exhibit a 16.32% reduction in TWR and a 26% decrease in OVC [13]. Additionally, MRR in micro-EDM is enhanced by 18.9% with coated electrodes. Among various micro-EDM electrode coatings, TiN, Ag, and ZrN, TiN demonstrates the highest efficiency [14]. For finishing applications, Gr electrodes coated with Cu via plating methods significantly reduce the SR of the machined surface [15]. Conversely, TiAlN coatings exhibit lower machining efficiency in micro-EDM compared to TiN coatings [16].

Given the divergent effects of various coating materials on electrode workability [17,18], a comprehensive evaluation of coating material effectiveness is imperative. While existing studies have explored the use of coated electrodes in micro-EDM, the body of knowledge remains limited. Therefore, validating the viability of micro-EDM with coated electrodes requires further research. This study meticulously explores the influence of carbon coating on WC electrode surfaces in micro-EDM applied to Ti–6Al–4V, examining their impact on depth of cut (Z), TWR, OVC, and post-machining surface quality. By addressing these knowledge gaps, this research contributes to the growing field of micro-EDM techniques and enhances the understanding of coated electrode applications.

## 2. Experimental Setup

The conclusive experimental trials were conducted using four distinct electrodes on Titanium Grade 5 plates with a thickness of 1.6 mm. The workpiece dimensions were 10 mm × 50 mm. Among the four electrodes, three were made of WC and were coated with AlCrN, Carbon, and TiN. Each set of the four different electrode configurations was assessed using the Hyper 10 EDM system. The experiments were performed using a micro-EDM apparatus (Manufacturer: Synergy Nano Systems, Vashi Navi Mumbai, India, Model: Hyper 10). The weight of each tool was measured both before and after each experiment using a precision analytical balance (Manufacturer: Ishida Co. Ltd., Kyoto, Japan, Model: DXR220). Preliminary experiments revealed that negative polarity settings did not yield favorable machining outcomes. The experiments incorporated three input parameters: Voltage, Capacitance, and RPM. Both coated and uncoated electrodes, characterized by a mean diameter, were employed for the final trials, as indicated in Table 1. The feed rate for the machine was set to 10 microns per minute. In micro-EDM, the dielectric fluid plays a crucial role during the machining operation, significantly influencing the machining characteristics. Various dielectrics, such as kerosene, deionized water, EDM oil, and hydrocarbon oil, affect performance criteria, including MRR, TWR, diameter variance at the entry and exit holes, and surface integrity during the machining of titanium alloy (Ti–6Al–4V). In this experimental work, EDM oil was used as the dielectric fluid, as detailed in Table 2.

**Figure 1:**
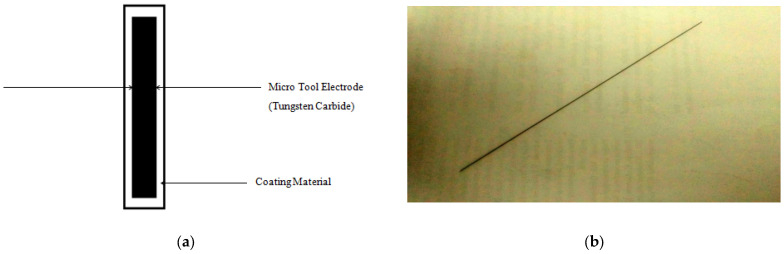
(**a**) Schematic representation of a thin film-coated tool electrode and (**b**) development of thin film coating of tin material on micro tool electrode.

In micro-EDM, a 0.5 mm diameter micro tool electrode was used. The schematic representation of a coated microtool electrode is shown in Figure 1. The microtool electrode includes a core of Tungsten Carbide with coatings of conductive materials such as TiN, AlCrN, and Carbon. The substrate (tool) diameter was 490 microns, uniformly coated with a 0.05 mm layer.

Coatings are applied to alter substrate properties such as corrosion and wear resistance, adhesion, and electrical conductivity. A major consideration in coating processes is uniform thickness. The primary objective of coating tools is to minimize TWR and extend tool life. In this study, TiN, AlCrN, and Carbon coatings were used.

**Figure 2:**
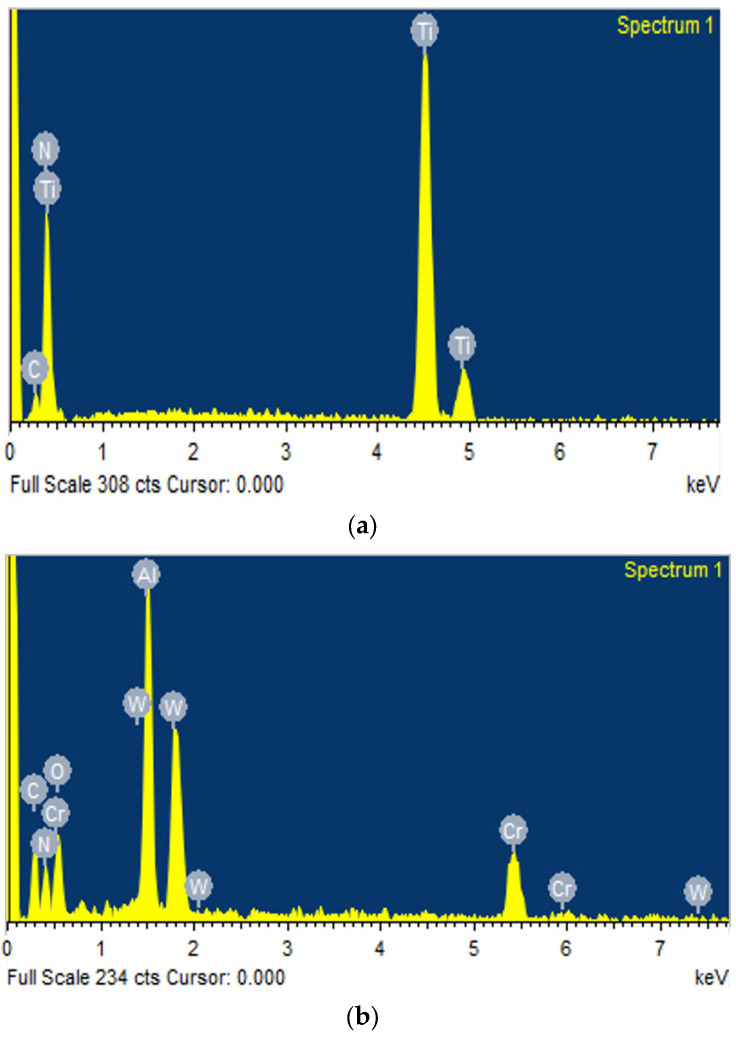

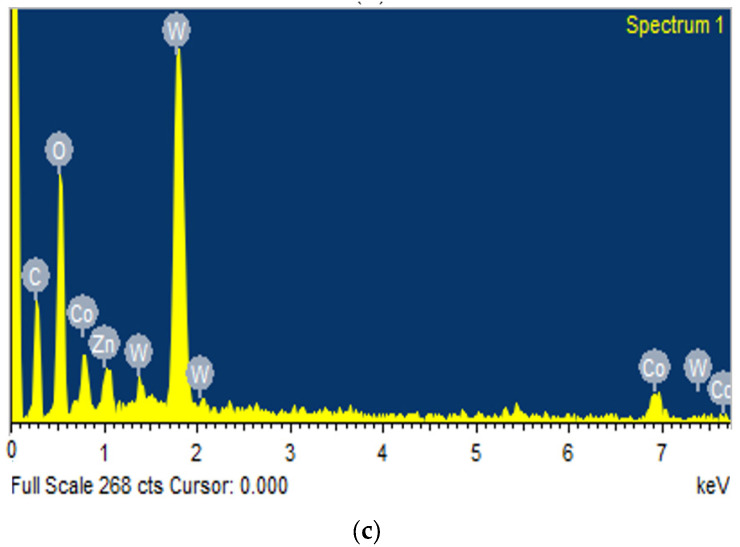
EDAX of Thin Film Coating. (**a**) Titanium Nitride. (**b**) Aluminum Chromium Nitride. (**c**) Carbon.

The development of the thin film coating of TiN was achieved using the Physical Vapor Deposition (PVD) method at Balzer India Pvt. Ltd., Pune. The elemental composition and properties of the coatings were analyzed using Energy-Dispersive X-ray Analysis (EDAX). Table 3 shows the weight and atomic percentages of the elements present in the coatings, and Figure 2 presents the EDAX analysis of the TiN coating, confirming the presence of titanium and nitride.

**Figure 3:**
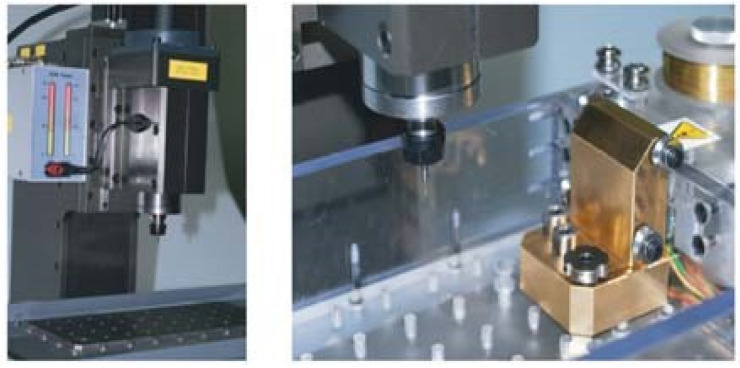
Hyper 10 Micro-EDM machine.

WC electrodes are known for their toughness, allowing them to drill through highly durable materials. They are also capable of drilling extremely small holes, with diameters down to 0.045 mm. WC electrodes with a mean diameter of 490 μm were used for the final experiments. Coatings of TiN (6.663 μm), Carbon (10 μm), and AlCrN (3.385 μm) were applied to the electrodes. A standardized duration of 15 min was maintained across all experiments, conducted under straight polarity conditions (also known as normal polarity, where the workpiece is positively charged while the tool (electrode) is negatively charged). The results of the experiments with both coated and uncoated electrodes are summarized in Table 4. Figure 3 illustrates the Hyper 10 micro-EDM machine.

## 3. Results and Discussion

### 3.1. Influence of Electrode Coating Material on Z Coordinate

**Figure 4:**
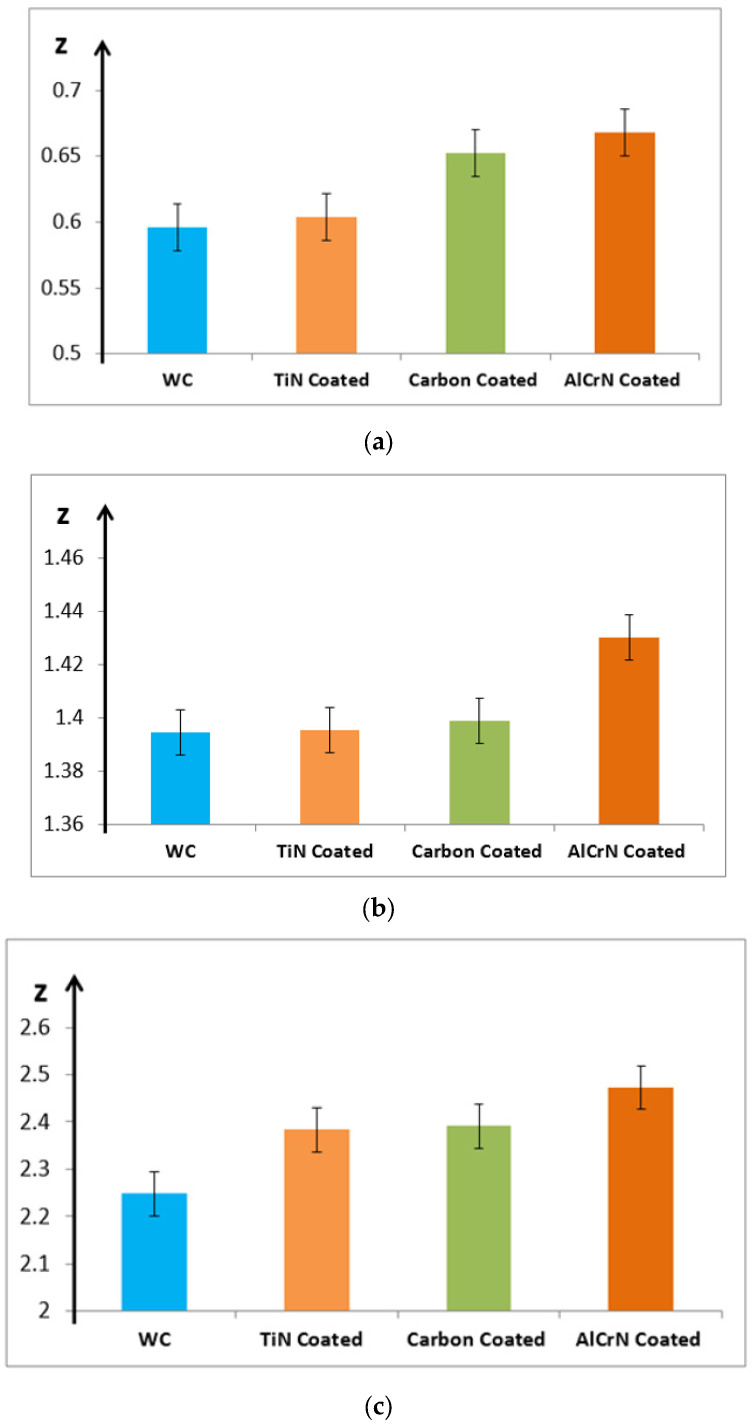
Influence of coating material on Z coordinate. (**a**) V = 120; C = 100 pF and RPM = 200 rpm. (**b**) V = 140; C = 1000 pF and RPM = 400 rpm. (**c**) V = 160; C = 10,000 pF and RPM = 600 rpm.

The Z coordinate, representing the electrode depth as indicated by the machine’s display, is significantly impacted by the electrode coating material. Figure 4 illustrates how different coatings influence machining productivity, particularly the depth of cut (Z). It is clear that the coating materials enhance Z values across various technological configurations. Specifically, AlCrN coating material achieves the highest Z values, followed by carbon and TiN coatings. The effect of coating materials on Z is contingent upon specific machining conditions.

Figure 4a shows that compared to the uncoated WC electrode, the TiN-coated electrode increases Z by 1.34%, the carbon-coated electrode by 9.41%, and the AlCrN-coated electrode by 12.05%. In Figure 4b, the TiN-coated electrode increases Z by 0.06%, the carbon-coated electrode by 0.31%, and the AlCrN-coated electrode by 2.53%. Figure 4c demonstrates that the TiN, carbon, and AlCrN-coated electrodes increase Z by 6.0%, 6.34%, and 9.97%, respectively, compared to the uncoated WC electrode. The variations in Z are most pronounced under the conditions depicted in Figure 4c, where Z reaches its maximum value. These results highlight the critical need to understand the influence of technological parameters on quality parameters in micro-EDM using coated electrodes.

### 3.2. Influence of Electrode Coating Material on TWR

Tool Wear Rate (TWR), calculated using Equation (1), indicates the amount of material removed from the tool:

(1) $$TWR\;\left(\frac{gm}{min}\right)=\frac{Wtb\;\left(gms\right)-Wta\;\left(gms\right)}{t\;\left(min\right)}$$

where Wtb is the tool’s weight before machining, Wta is the tool’s weight after machining, and t is the machining time.

**Figure 5:**
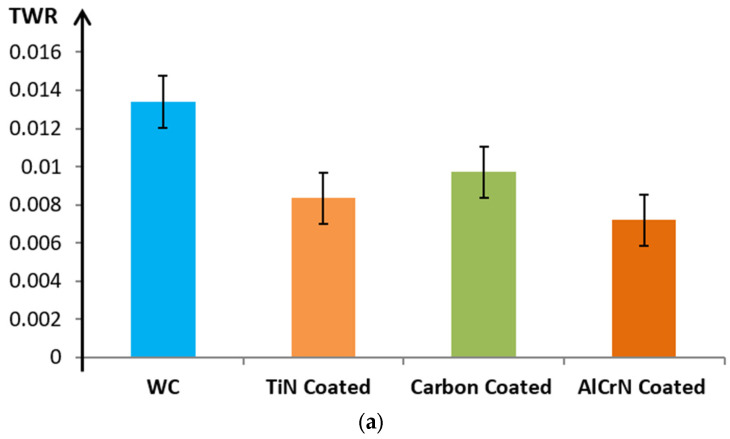

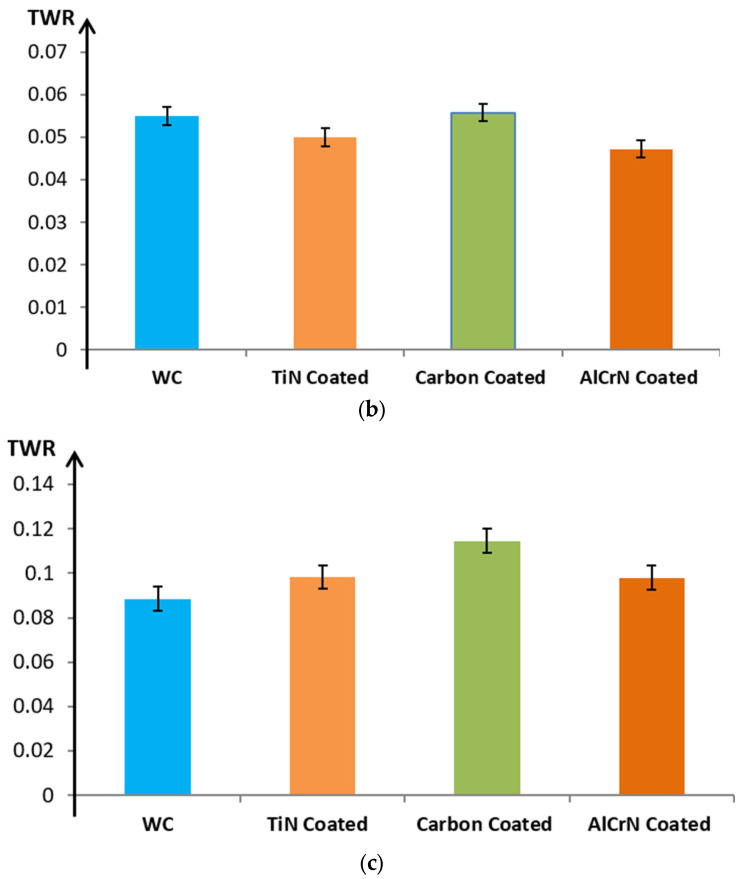
Influence of coating material on TWR. (**a**) V = 120; C = 100 pF and RPM = 200 rpm. (**b**) V = 140; C = 1000 pF and RPM = 400 rpm. (**c**) V = 160; C = 10,000 pF and RPM = 600 rpm.

Figure 5 shows that the AlCrN-coated electrode exhibits the lowest TWR, followed by the TiN-coated and carbon-coated electrodes. The superior wear resistance of the AlCrN coating likely accounts for this reduction in TWR. As depicted in Figure 5a, the TWR of the AlCrN-coated WC electrode experiences a notable reduction of 46.19% compared to its uncoated counterpart. Similarly, the TiN-coated electrode witnesses a TWR decline of 37.76%, whereas the carbon-coated electrode records a decrease of 27.46% in TWR. In contrast, Figure 5b demonstrates increased TWR for both coated and uncoated WC electrodes. Specifically, the TWR of the AlCrN and TiN-coated electrodes decreases by 14.18% and 9.09%, respectively, compared to the uncoated WC electrode, while the carbon-coated electrode exhibits a 1.45% increase in TWR.

Moreover, Figure 5c accentuates that the TWR of coated electrodes significantly exceeds that of uncoated electrodes, suggesting that using coated electrodes under these specific experimental conditions may not be appropriate. More precisely, Figure 5c illustrates that, in comparison to the TWR of uncoated electrodes, TWR values for all AlCrN, TiN, and carbon-coated electrodes increase by 10.75%, 11.20%, and 29.53%, respectively.

These findings suggest that AlCrN-coated electrodes effectively reduce TWR, rendering them suitable for applications requiring enhanced wear resistance. Conversely, carbon coatings may exhibit diminished effectiveness under the tested conditions.

These preliminary results emphasize the importance of investigating the intricate relationship between technological parameters and the TWR of coated electrodes. Such investigation is crucial for optimizing the effectiveness of coatings in improving electrode wear resistance in micro-EDM [17,18], thus directly contributing to enhanced machining precision. A thorough understanding of coating material properties is essential for a more comprehensive elucidation of their impact on EDM quality criteria [19,20,21]. Subsequent research efforts stand to benefit from refining these preliminary findings and delving deeper into the underlying mechanisms governing these effects.

### 3.3. Impact of Electrode Coating Material on OVC

Overcut: OVC is calculated as Equation (2).

(2) $$\mathrm{OVC}=\frac{D\;\left(Diameter\;of\;Workpiece\;Hole\right)-d\;\left(Diamter\;of\;Electrode\right)}{2}$$

**Figure 6:**
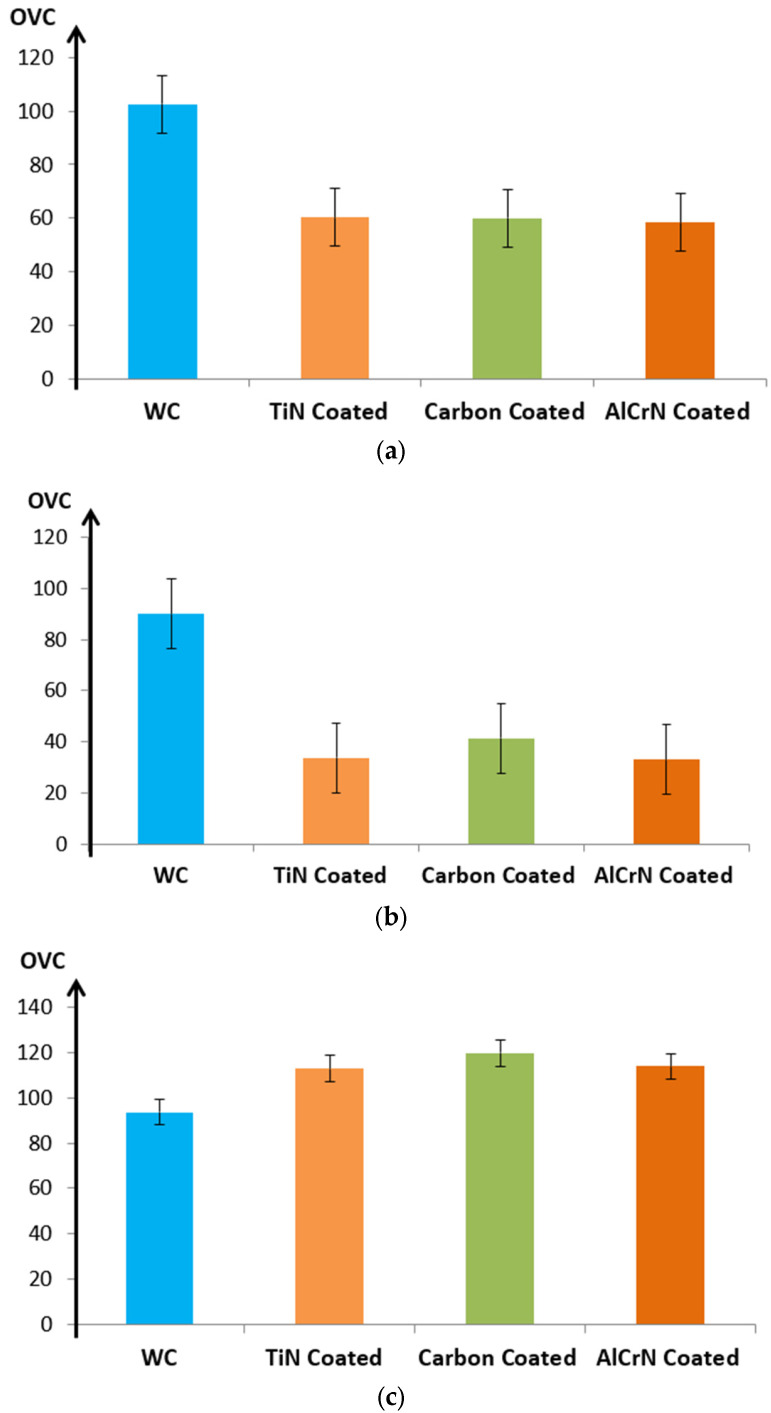
Influence of coating material on Overcut (OVC). (**a**) V = 120; C = 100 pF and RPM = 200 rpm. (**b**) V = 140; C = 1000 pF and RPM = 400 rpm. (**c**) V = 160; C = 10,000 pF and RPM = 600 rpm.

OVC directly affects machining precision. As OVC increases, the gap between the electrode and the machined surface widens, resulting in decreased machining precision. This phenomenon highlights that higher OVC values and variations correspond to diminished accuracy in machining. The impact of coating material on OVC under various micro-EDM conditions is depicted in Figure 6.

Figure 6a,c show that coated electrodes result in significantly reduced OVC compared to uncoated electrodes. For example, in Figure 6a, the AlCrN-coated electrode reduces OVC by 43.09%, TiN by 41.25%, and carbon by 41.43%. Figure 6b reflects similar trends, with OVC reductions of 63.37%, 62.44%, and 54.12% for AlCrN, TiN, and carbon coatings, respectively. However, Figure 6c shows an increase in OVC for coated electrodes by 21.59%, 20.60%, and 27.83% for AlCrN, TiN, and carbon coatings, respectively, compared to the uncoated WC electrode.

The research findings underscore that setups similar to those depicted in Figure 6a,b hold promise for enhancing machining accuracy [22]. This emphasizes the necessity for an optimized set of technological parameters when utilizing coated electrodes to achieve better results in micro-EDM processes.

### 3.4. Analysis of Machining Quality Post Micro-EDM

**Figure 7:**
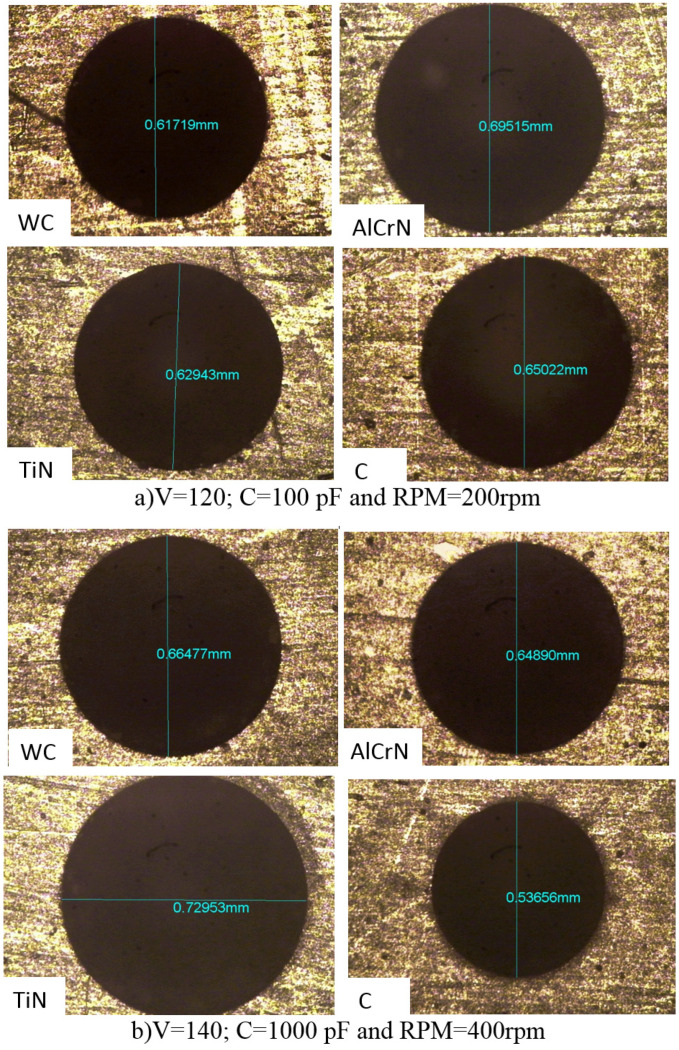

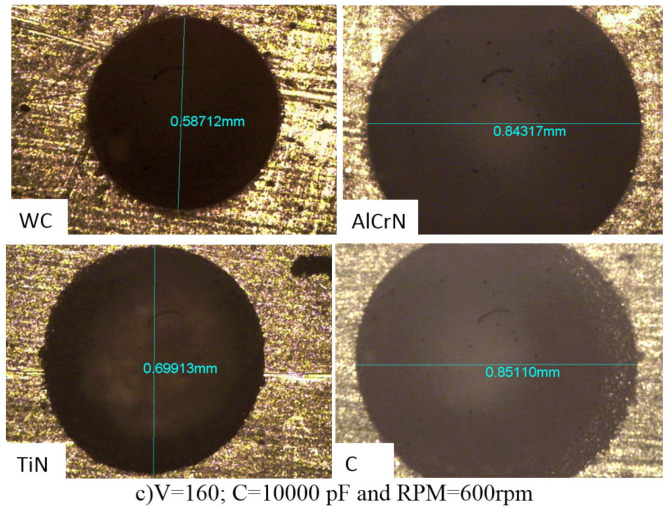
Machining dimensions in micro-EDM.

**Figure 8:**
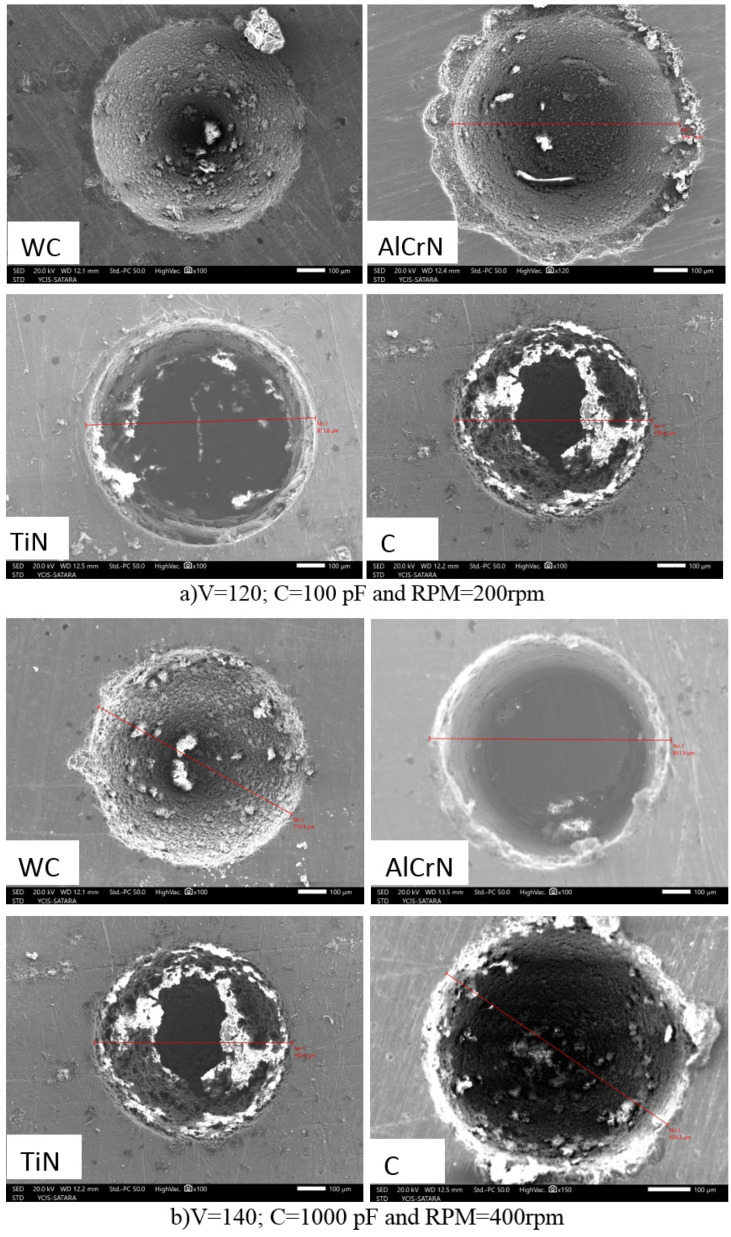

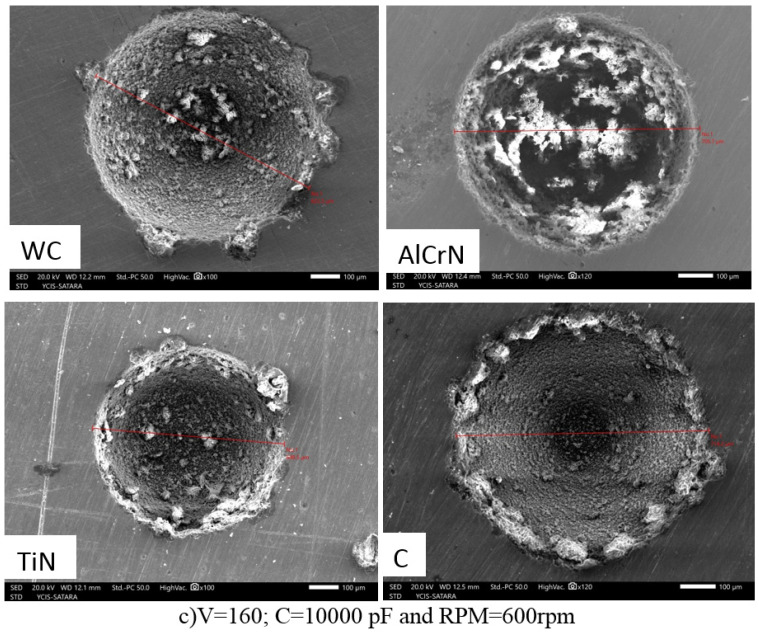
SEM imagery of the machined surface in micro-EDM.

**Figure 9:**
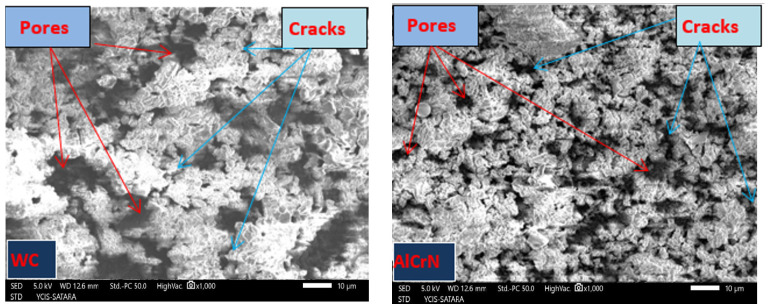

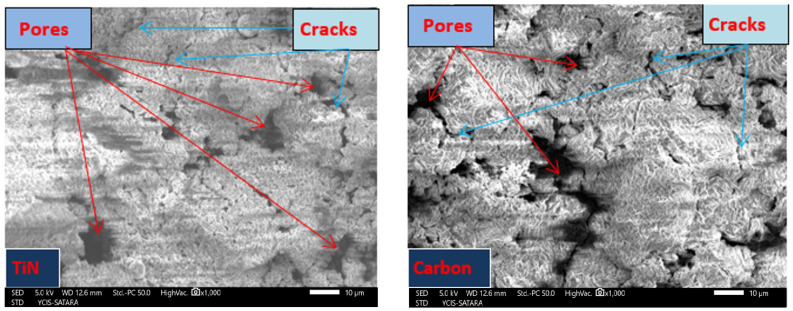
Topography of the machined Surface after micro-EDM (V = 120; C = 100 pF and RPM = 200 rpm).

**Figure 10:**
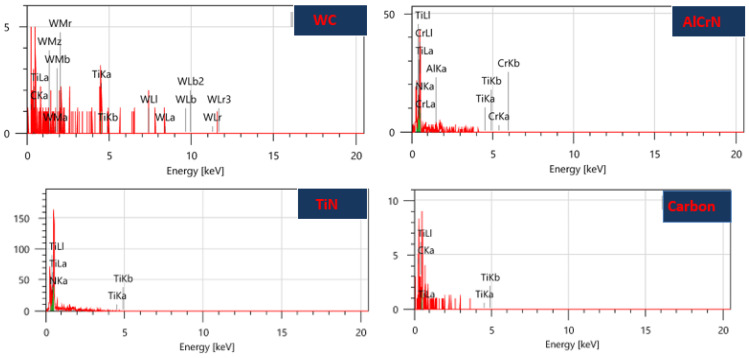
EDX analysis of the machined surface after micro-EDM. (V = 120; C = 100 pF and RPM = 200 rpm).

Figure 7, Figure 8, Figure 9 and Figure 10 evaluate machining quality post-micro-EDM using both coated and uncoated electrodes. Figure 7 presents the machined hole dimensions, showing satisfactory results. Figure 8’s SEM images reveal chip particle adhesion to the machined hole’s surface, with varying densities across different conditions. Figure 9 displays the machined surface topography, characterized by micro-scale voids and cracks due to thermal residual stress and the impact of electrical sparks. Figure 10’s EDX (Energy-Dispersive X-ray Spectroscopy) analysis confirms the presence of coating material elements on the machined surface, suggesting potential benefits such as improved mechanical properties.

Figure 8 presents SEM images illustrating the significant adhesion of chip particles to the machined hole’s surface, with chip density escalating toward the hole’s base. Comparative analysis reveals varying levels of particle adherence, with Figure 8c exhibiting the highest density and Figure 8b the lowest. The adhesion of particles to the machined surface is attributed to the rapid cooling of melted and evaporated workpieces and electrode materials by the dielectric solution [16].

Figure 9 showcases microscopic voids and cracks characterizing the topography of the machined surface [17]. Micro-cracking is ascribed to thermal residual stress induced during micro-EDM [18,19], while craters and micro-voids result from the impact of electrical sparks and gas bubble formation during machining, respectively. These surface irregularities significantly affect the surface’s workability, gloss, wear, and fatigue strength.

The EDX analysis of the machined surface (Figure 10) reveals that material from the electrode’s surface layer is present on the machined surface. Specifically, the W element is found exclusively on the surface when a WC-coated electrode is used, whereas alloying elements from the coating material are detected on the machined surfaces when using other coated electrodes. This dispersion of thin film coating material on the surface is attributed to the high temperature during pulse on time, enhancing the coating’s mechanical properties. Additionally, the presence of Al, Cr, and N elements suggests potential advantages such as improved mechanical properties and the potential to replace costly surface spray methods like PVD, PVC, etc. The protective role of the coating on the base electrode during machining contributes to enhanced working conditions of the electrode in micro-EDM [23,24].

### 3.5. Evaluation of Research Results and Direction for Future Research

This research demonstrates the benefits of using coated electrodes in micro-EDM. The findings show significant improvements in TWR and OVC, enhancing machining accuracy and the durability of tool electrodes. Future research should refine these findings and examine the interactions between process parameters and electrode coatings to optimize micro-EDM efficiency.

Table 5 compares the obtained results with those reported in the literature [12,19,25], illustrating the advantages of optimizing micro-EDM with coated electrodes. Future studies should delve deeper into the properties of coating materials and their impact on machining quality to further enhance micro-EDM applications. Additionally, systematic exploration of multi-objective optimization techniques will further improve machining outcomes in micro-EDM.

## 4. Conclusions

Extensive investigation into quality parameters within the realm of micro-EDM, involving both coated and uncoated electrodes for the precision machining of titanium alloy (Ti–6Al–4V), has been systematically conducted. Derived from the empirical findings, the subsequent conclusions are drawn:

- Enhancement of Z and Machining Productivity: The introduction of surface coating materials onto the electrode (TiN, AlCrN, and C) has exhibited a substantial elevation in Z. This marked enhancement has, in turn, led to a notable augmentation in the machining productivity when employing coated electrodes within micro-EDM;
- Uniformity in Impact on OVC and TWR: The impact of electrode material has shown noteworthy parallelism in its influence on both OVC and TWR. Notably, the degree of improvement in TWR and OVC is contingent upon the selected technological parameter levels within the ambit of micro-EDM, as dictated by the study;
- Impact on Machined Surface Quality and Geometric Precision: The quality of the machined surface and the precision of the resultant workpiece shape in micro-EDM are significantly affected by diverse electrode types and the prevailing machining conditions. The increased adhesion of particles towards the lower extremities of the machined hole surface can be attributed to the higher temperature gradients and rapid cooling in these regions, causing re-solidified material to adhere more readily;
- Sensitivity to Control Technology Parameters: While the study suggests a potential sensitivity of quality parameters to control technology parameters, further empirical evidence is needed to substantiate this claim conclusively. This highlights the necessity for directed investigations to ascertain the optimal and judicious technological parameters in the context of micro-EDM employing coated electrodes;
- Avenues for Further Exploration: Subsequent research endeavors should be directed towards a comprehensive analysis of coating material thickness and its endurance on the functionality of coated electrodes. Additionally, exploration into augmenting the mechanical properties of the machined surface layer via penetration of the coating material subsequent to micro-EDM involving coated electrodes warrants careful examination.

The cumulative outcomes of this research substantiate the intricate interplay between electrode coatings, machining parameters, and resultant quality metrics in the context of micro-EDM. These insights underscore the necessity for ongoing and systematic investigations to propel advancements in micro-EDM methodologies, thereby yielding refined machining outcomes and enhanced functional properties for applications involving Ti–6Al–4V materials.

## Figures and Tables

**Table 1:** Electrodes utilized in final experiments.

Sr. No.	Materials of Tool	Specifications	Diameter (μm)
1	Tungsten Carbide Electrode	Non-Coated	490
2	Titanium Nitride	Coated	490 + 6.663 × 2 = 503.326
3	Carbon-coated Electrode	Coated	490 + 10 × 2 = 510
2	Alchrona-coated Electrode	Coated	490 + 3.385 × 2 = 496.77

**Table 2:** Properties of dielectric fluid.

Properties	Values
Specific gravity	81
Flashpoint (°C)	100
Viscosity (mm^2^/s)	2.53
Appearance	Colorless transparent liquid

**Table 3:** Elements in micro-electrode coating.

Element	Weight%	Atomic%
**Titanium Nitride Coating**		
C K	5.25	11.53
N K	27.21	51.26
Ti K	67.54	37.21
**Aluminum Chromium Nitride Coating**		
C K	16.09	31.98
N K	15.79	26.91
O K	13.00	19.40
**Carbon Coating**		
C K	19.06	42.54
O K	27.60	46.24
Co K	8.06	3.66
Zn L	3.64	1.49
W M	41.64	6.07

**Table 4:** Experimental results.

Process Parameters			Response Variables											
			Tungsten Carbide Electrode			TiN-Coated Microtool Electrode			Carbon-Coated Microtool Electrode			AlCrNi-Coated Microtool Electrode		
Voltage (V)	Capacitance (pF)	RPM (rpm)	Z (mm)	TWR (mg/min)	Overcut (μm)	Z (mm)	TWR (mg/min)	Overcut (μm)	Z (mm)	TWR (mg/min)	Overcut (μm)	Z (mm)	TWR (mg/min)	Overcut (μm)
120	100	200	0.596	0.0134	102.575	0.604	0.00834	60.2635	0.6521	0.00972	60.079	0.6678	0.00721	58.3791
140	1000	400	1.3947	0.055	90.02	1.3956	0.05	33.8096	1.399	0.0558	41.2972	1.43	0.0472	32.9744
160	10,000	600	2.2485	0.0884	63.59	2.3833	0.0983	112.8685	2.3911	0.1145	119.632	2.4727	0.0979	113.7922

**Table 5:** Comparison between obtained and reported literature results.

Comparison Results		Process Parameter	Quality Criteria		
			Z (mm)	TWR (mg/min)	OVC (µm)
Obtained Results	TiN-coated	V = 160 V, C = 10,000 pF, and RT = 600 rpm	2.3833	0.0983	112.8685
	Carbon-coated		2.3911	0.1145	119.632
	AlCrN-coated		2.4727	0.0979	113.7922
Reported Literature Results: Optimizing micro-EDM with Coated Electrodes	[12]	V = 120 V, C = 10,000 pF, and RT = 600 rpm	2.07	-	59.23
	[19]	V = 140 V, C = 10,000 pF, and RT = 200 rpm	2.92	0.05	45.19
	[25]	V = 160 V, C = 10,000 pF, and RT = 600 rpm	2.496	0.0875	-

## Data Availability

The original contributions presented in the study are included in the article, further inquiries can be directed to the corresponding author.
